# Andrographolide contributes to the attenuation of cardiac hypertrophy by suppressing endoplasmic reticulum stress

**DOI:** 10.1080/13880209.2022.2157021

**Published:** 2022-12-22

**Authors:** Qingxin Tian, Jianlong Liu, Qin Chen, Mingxiao Zhang

**Affiliations:** a Department of Anesthesiology, The First Affiliated Hospital of Wenzhou Medical University, Wenzhou, China; b Intensive Care Unit, The First Affiliated Hospital of Wenzhou Medical University, Wenzhou, China

**Keywords:** Transverse aortic constriction, angiotensin II, heart failure, pressure overload, cardiac fibrosis

## Abstract

**Context:**

Andrographolide (Andr) is a bioactive Andr diterpenoid extracted from herbaceous *Andrographis paniculata* (Burm. F.) Wall. ex Nees (Acanthaceae). Andr can relieve cardiac dysfunction in mice by inhibiting the mitogen-activated protein kinases (MAPK) pathway.

**Objective:**

This study investigates the efficacy and underlying mechanism of Andr on cardiac hypertrophy in mice.

**Materials and methods:**

Male C57 mice (20–25 g, 6–8 weeks) were divided into four groups (*n* = 10 mice/group) as sham group (sham operation), transverse aortic constriction (TAC) model group, TAC + Andr 100 mg/kg group and TAC + Andr 200 mg/kg group. Andr groups were given intragastric administration of Andr (100 and 200 mg/kg) once a day for 14 consecutive days. An *in vitro* hypertrophy model was established by adding 1 μM of Ang II to H9c2 cells for 48 h induction.

**Results:**

In TAC-mice, Andr improved echocardiographic indices [reduced LVESD (30.4% or 37.1%) and LVEDD (24.8% or 26.4%), increased EF (22.9% or 42.6%) and FS (25.4% or 52.2%)], reduced BNP (11.5% or 23.6%) and Ang II levels (10.3% or 32.8%), attenuates cardiac fibrosis and reduces cardiac cell apoptosis in TAC mice. *In vitro,* Andr attenuated cardiomyocyte hypertrophy and decreased the protein expression of GRP78 (67.8%), GRP94 (47.6%), p-PERK (44.9%) and CHOP (66.8%) in Ang-II-induced H9c2 cells and reversed after endoplasmic reticulum (ER) stress agonist Tunicamycin (TN) treatment.

**Discussion and conclusions:**

Andr was found to be an anti-hypertrophic regulator, which could attenuate cardiac hypertrophy by suppressing ER stress. It may be a new therapeutic drug for cardiac hypertrophy.

## Introduction

Cardiac hypertrophy refers to a response of the heart to augmented workload, such as dilated cardiomyopathy, aortic stenosis and hypertension (Myers et al. 2017). Pathological cardiac hypertrophy is characterized by enlarged cardiomyocytes and obstructed contractility of the heart, accompanied by internal changes in cardiomyocytes, including apoptosis, cardiac fibrosis, metabolism and gene expression (Tham et al. 2015; Gesmundo et al. 2017). In the case of coronary artery disease, valvular heart disease and hypertension, the heart may also become hypertrophic under chronic stress or volume overload (Weeks and McMullen 2011). Moreover, cardiac hypertrophy has been reported to usually develop into heart failure (Shimizu and Minamino 2016). Several researchers have clarified that multiple signalling pathways are involved in the occurrence of cardiac hypertrophy, such as typical G protein-coupled receptor (GPCR) and calcineurin-activated T nuclear factor (NFAT) signalling pathways (Madukwe et al. 2018; Zhang et al. 2020). Nevertheless, the mortality from heart failure remains high due to the complex mechanism of the transition from hypertrophy to heart failure, as well as the difficulty of reversing cardiac hypertrophy. Therefore, it is urgent to find a new pharmacological drug that can suppress the progression of cardiac hypertrophy.

As the main signal transduction organelle, endoplasmic reticulum (ER) is very sensitive to changes in cell homeostasis and function. Accumulating evidence suggests that many physiological and pathological conditions such as oxidative stress, hypoxia, gene mutations and heart shock can interfere with protein folding in ER, leading to ER stress (Peters et al. 2015). To our knowledge, there are three ER stress sensors, including activated transcription factor 6 (ATF6), ER-resident PKR-like eIF2α kinase (PERK) and inositol-requiring 1 (IRE1). The sensors determine the accumulation of misfolded or unfolded protein at the beginning of ER stress and trigger unfolded protein response (UPR) when the ER quality control system cannot handle these excessive proteins (Liu et al. 2015). Importantly, PERK is activated under ER stress conditions. An earlier study pointed out that PERK is required to protect the heart from pressure overload-induced congestive heart failure (Liu et al. 2014). Also, previous research has indicated that ER stress and its mediated apoptosis are closely associated with the development and progression of cardiac hypertrophy (Rani et al. 2017). Therefore, alleviating ER stress may be beneficial to cardiac hypertrophy treatment.

Andrographolide (Andr), a bioactive Andr diterpenoid extracted from herbaceous *Andrographis paniculata* (Burm. F.) Wall. ex Nees (Acanthaceae), is traditionally utilized to treat cough, diarrhoea, fever and tuberculosis (Banerjee et al. 2017). Andr is identified as one of the leading candidates for the treatment of malignancy by blocking JNK-signal transducers and transcriptional activators (Islam et al. 2018), leading to suppression of cancer cell proliferation, survival and metastasis. In addition, Andr can relieve cardiac dysfunction in mice by inhibiting the mitogen-activated protein kinases (MAPK) pathway (Wu et al. 2017). However, the impact of Andr and its underlying mechanism on cardiac hypertrophy remain obscure.

This study attempted to test the hypothesis that Andr can attenuate cardiac fibrosis and reduce cardiac cell apoptosis, thereby alleviating cardiac hypertrophy in mice by suppressing ER stress.

## Materials and methods

### Animals

A total of 40 male C57 mice aged 6–8 weeks and weighing 20–25 g were purchased from Shanghai SLACCAS Laboratory Animal Co. (Shanghai, China) and housed at Wenzhou Medical University (Wenzhou, China) under controlled temperature and humidity. They were randomly divided into the sham group (sham operation), transverse aortic constriction (TAC) model group, TAC + Andr 100 mg/kg group and TAC + Andr 200 mg/kg group, with 10 mice per group. Protocols involving the use of the animals were approved by the Experimental Animal Ethics Committee of Zhejiang Haikang Biological Products Co (Protocol number: HKSYDWLL2020012). Andr was extracted from plant material from Shanghai Winberb, China, with a purity >98 which was detected by High-Performance Liquid Chromatography (HPLC).

### Establishment of TCA model in mice

After one week of acclimatization, mice were anaesthetized *via* intraperitoneal injection of 50 mg/kg sodium pentobarbital before surgery. Briefly, the skin was cut 0.5–1 cm vertically along the middle line of the neck and chest of the mice using ophthalmic scissors, and the muscle was split from the second ribs and the third frame (5 mm) using ophthalmic forceps. Next, the thymoma lobes were separated using micro tweezers to allow clear visualization of the aortic arch and the two carotid arteries, and then a 6-0 nylon suture was carefully pulled between the left common carotid artery and the right indolent artery, and below the aortic arch. After that, a self-made 26 G pad needle was placed near the aortic arch to tie and fix the aortic arch. Three knots were tied to ensure the ligation was stable, and the pad needle was carefully removed. After the successful establishment of TCA, marked enhancement of the right common carotid pulse was observed. Finally, the wound and skin were sutured. It was worth noting that mice in the sham group underwent thoracotomy only and were not ligated.

From the day of modelling, mice in the TAC + Andr groups were given intragastric administration of Andr (100 and 200 mg/kg) once a day for 14 consecutive days. Mice in the model and sham groups were given the same volume of distilled water. After the experiment, the weight of the mice was measured. Heart index = heart weight/body weight.

### M-mode echocardiography

Two weeks after TAC surgery, cardiac functions were measured *via* echocardiography as previously described (Wu et al. 2017). M-mode echocardiography was applied to measure left ventricular end-systolic diameter (LVESD) and left ventricular end-diastolic diameter (LVEDD), as well as LV ejection fraction (EF) and LV shortening score (FS). Each animal was sampled three times and the mean values of three cardiac cycles were recorded.

### ELISA assay

Blood samples were collected from the eyeballs of mice (venous sinus) after anaesthesia and centrifuged at 1500 rpm for 10 min. The plasma levels of brain natriuretic peptide (BNP) and angiotensin II (Ang II) were detected using the enzyme-linked immunosorbent assay (ELISA) kit according to the manufacturer’s protocol (Beijing Chenglin Biotechnology, China).

### Histopathological analysis

After euthanasia, the hearts of mice were quickly removed through a chest incision. Blood clots in the heart cavity were rinsed with PBS and the connective tissue and blood vessels around the heart were cut off. Then, the heart mass was measured to calculate the heart index. Heart index = heart mass (g)/weight (g). After PFA fixation of the hearts of five mice from each group, pathological tests were performed. The hearts of the other five mice were stored at −80 °C after quick freezing.

Hematoxylin and eosin (H&E) staining and Masson’s trichrome staining were adopted to measure the cardiomyocyte size and detect fibrosis, respectively. The cardiac tissues from each group were fixed in 4% paraformaldehyde at 4 °C for 24 h and then embedded in paraffin. Next, the paraffin-embedded sections of cardiac tissues were stained with an H&E staining kit (Beyotime, China) or a Masson staining kit (Beijing Solarbio Science & Technology Co., Ltd., China). Finally, the histopathological examination of the lesions was performed under a light microscope.

### TUNEL staining

To determine apoptosis, the TdT-mediated-dUTP nick-end labelling (TUNEL) assay was carried out. Heart sections of mice in each group were stained with the TUNEL Apoptosis Detection Kit (Roche Diagnostics, USA) and the images were observed under a microscope. Five high-power fields of each paraffin section were randomly selected. TUNEL positive nuclear showed blue fluorescence.

### Cell culture and treatment

H9c2 cells (Shanghai Cell Bank of the Chinese Academy of Science, China) were cultured in DMEM containing 10% foetal bovine serum and 100 μg/mL penicillin-streptomycin under 5% CO_2_ at 37 °C. Then, they were seeded on a six-well plate at 5 × 10^5^ cells/well. To simulate hypertrophy, 1 μM Ang II (Sigma, USA) was added to the cells and cultured in the medium for 48 h. The cells were then divided into five groups, namely, the control group, the Ang II group, three Ang II + Andr (1, 5 and 10 μM) groups and Ang II + 10 μM Andr + 10 μM ER stress agonist Tunicamycin (TN).

### Cell counting kit-8 assay

H9c2 cardiomyocytes were treated with Andr (0, 0.1, 0.5, 1, 5, 10, 25, 50, 100 and 250 μM) for 4 h. Cell viability was determined by the CCK-8 method in line with the manufacturer’s protocols (Wu et al. 2017), and the appropriate concentration was selected for modelling.

### Real-time PCR

Total RNA was extracted from the cultured H9c2 cells after treatment with TRIzol reagent (Invitrogen). Then, a reverse transcription kit was used to synthesize cDNA following the manufacturer’s protocols. PCR amplification was performed using a LightCycler 480 SYBR Green Master Mix (Roche, Mannheim, Germany) and standardized for β-actin. The mRNA expression of BNP, β-myosin heavy chain (β-MHC) and atrial natriuretic peptide (ANP) were measured using a real-time PCR system, and the primer sequences were shown as follows: BNP (forward primer, 5′-TGATTCTGCTCCTGCTTTTC-3′; reverse primer, 5′-GTGGATTGTTCTGGAGACTG-3′), β-MHC (forward primer, 5′-GCTGAAAGCAGAAAGAGATTATC-3′; reverse primer, 5′-TGGAGTTCTTCTCTTCTGGAG-3′), ANP (forward primer, 5′-ATCTGATGGATTTCAAGAACC-3′; reverse primer, 5′-CTCTGAGACGGGTTGACTTC-3′) and β-actin (forward primer, 5′-TTGCTGACAGGATGCAGAAGGAGA-3′; reverse primer, 5′-ACTCCTGCTTGCTGATCCACATCT-3′).

### Immunofluorescence staining

Cells were fixed in 4% paraformaldehyde at room temperature for 10 min. After blocking with PBS containing 0.1% Triton-x and 1% PBS for 5 min, cells were incubated with a monoclonal antibody against F-actin (1:200, Merck Millipore, USA) overnight at 4 °C, followed by further incubation with the secondary antibody for 30 min at room temperature. Then, the sections were sealed using DAPI. Images of visible cells were observed under a fluorescence microscope (Zhang et al. 2019).

### Western blot

Western blot was used to detect the protein expression of cardiac hypertrophy-related markers (ANP, BNP and β-MHC) and ER stress-related markers [glucose-regulated protein 78 (GRP78), glucose-regulated protein 94 (GRP94), total (T)-PERK, phosphorylated (p)-PERK and CCAAT/enhancer binding protein homologous protein (CHOP)] in H9c2 cells. The cells were divided into the control, Ang II, Ang II + Andr and Ang II + Andr + TN groups. Cells in the Ang II + Andr group were treated with 10 μM Andr and those in the Ang II + Andr + TN group were administrated with 10 μM Andr and 10 μM TN. Total protein isolated from heart tissues and H9c2 cells was collected using cell lysis buffer, and the concentration of total protein was quantified by the BCA Protein Assay kit (Beyotime, China). After SDS electrophoresis and transfer to polyvinylidene fluoride membrane, the blots were blocked with 5% nonfat milk in 0.1% Tween 20 (TBST) for 2 h at room temperature. Then, primary antibodies against BNP, β-MHC, TGF-β, Bcl-2, Bax, GRP78, GRP94, ERO-1α, pELF-2α, ATF-4, CHOP and β-actin were incubated on the membrane at 4 °C overnight, followed by incubation with a secondary antibody coupled with horseradish peroxidase for 1 h. Finally, the protein bands were visualized with the chemiluminescence system and quantified *via* Image J computer software.

### Statistical analysis

All data were expressed as mean ± standard deviation (SD) using GraphPad Prism v8 software. One-way ANOVA and *post hoc* Tukey test were performed to evaluate the anti-hypertrophic effects of Andr *in vivo* and *in vitro*. A *p* value < 0.05 was considered statistically significant.

## Results

### Andr decreased cardiac dysfunction in mice with TAC

**Figure 1.**
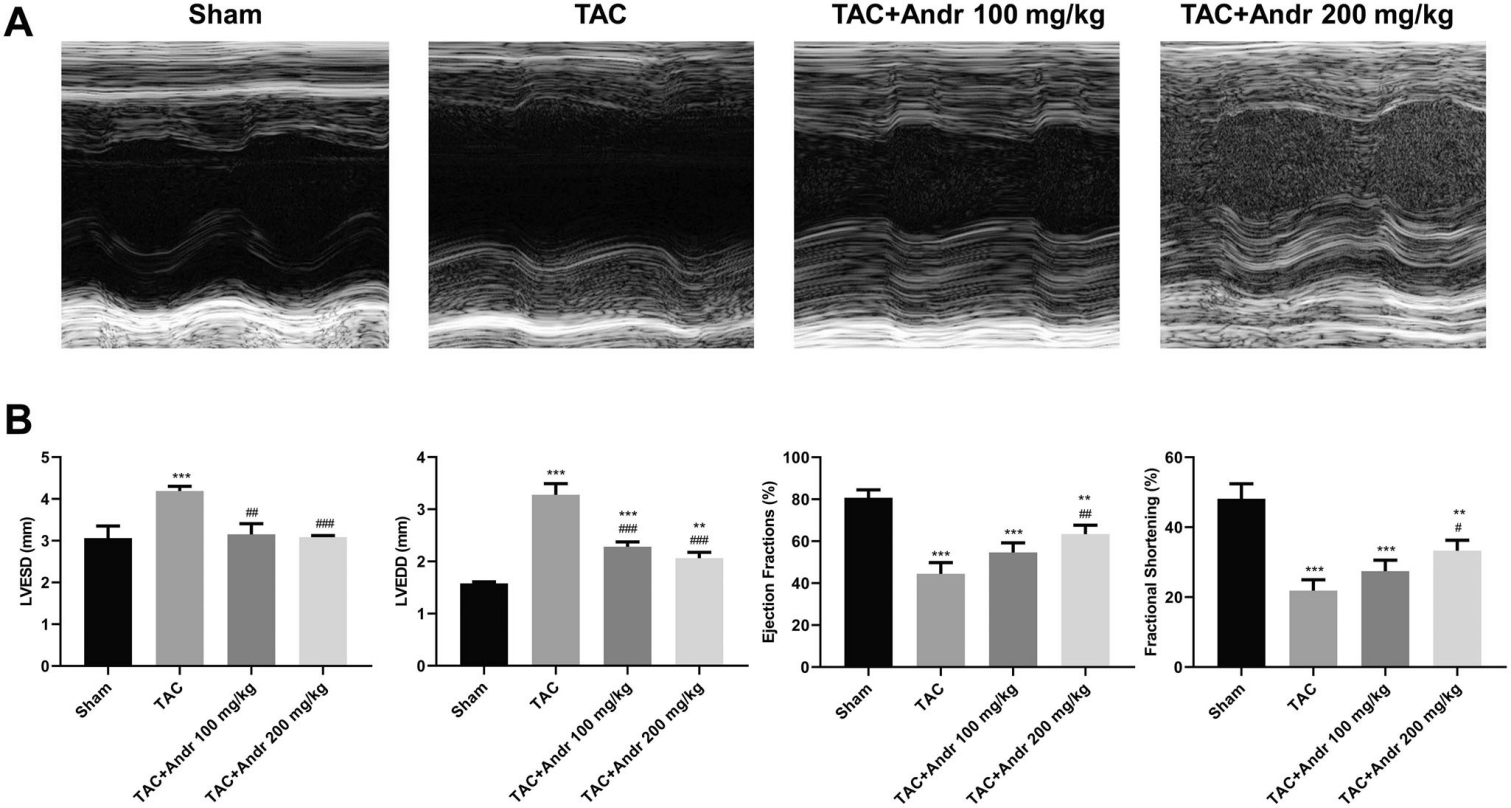
Andr reduces cardiac dysfunction *in vivo*. (A) Representative M-mode echocardiography of left ventricular chamber in the sham, TAC, TAC + Andr 100 mg/kg and TAC Andr 200 mg/kg groups. (B) Quantification of echocardiography parameters of left ventricular-end-systolic diameter (LVESD), left-ventricular-end diastolic diameter (LVEDD), LV ejection fraction (EF) and LV shortening score (FS) at different groups of mice. **p < 0.01 and ***p < 0.001 vs. the sham group; ^#^*p* < 0.05, ^##^*p* < 0.01 and ^###^*p* < 0.001 vs. the TAC group.

To evaluate the efficacy of Andr on cardiac contractile function *in vivo*, mice were given intragastric administration of Andr (100 and 200 mg/kg). As shown in Figure 1(A,B), heart function was significantly deteriorated, especially systolic function two weeks after TAC surgery. Conversely, Andr prominently ameliorated left ventricular contractile function as reflected by decreased LVESD and LVEDD and enhanced left ventricular EF and FS compared to the TAC group. These data indicated that Andr could attenuate cardiac damage in TAC mice.

### Andr inhibits cardiac hypertrophy in TAC model

**Figure 2.**
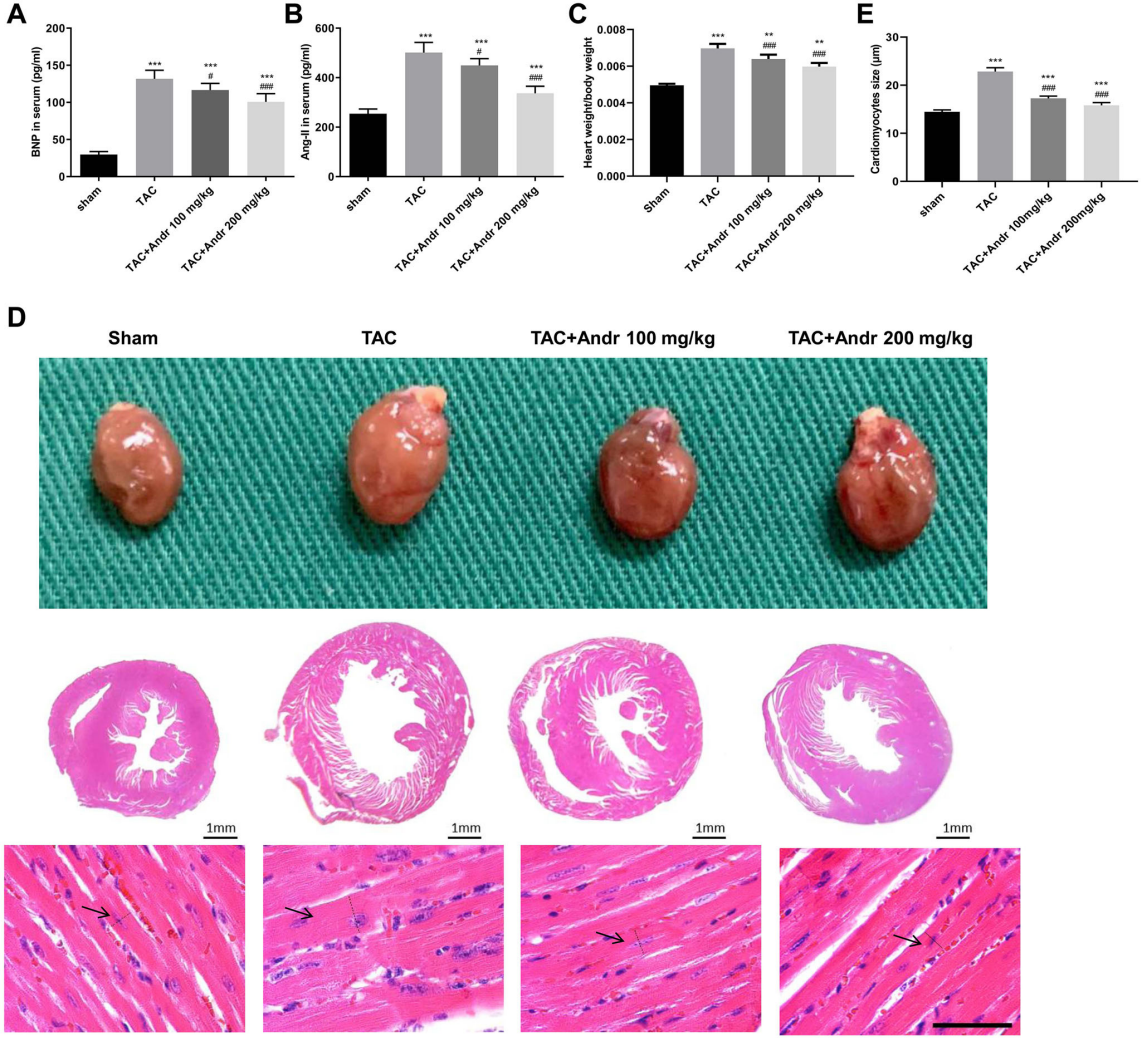
Andr inhibits cardiac hypertrophy in TAC mice. Serum levels of (A) brain natriuretic peptide (BNP) and (B) angiotensin II (Ang II) were determined by ELISA assay. (C) The heart weight to body weight ratios of mice in different groups. (D) Representative images of gross hearts (first panel) and H&E staining of hearts at scale bar = 1 mm (second panel) and scale bar = 50 μm (third panel) from each group of mice. (E) Quantification of cardiomyocyte size of the indicated groups. **p < 0.01 and ***p < 0.001 vs. the sham group; ^#^*p* < 0.05 and ^###^*p* < 0.001 vs. the TAC group.

As shown in Figure 2(A,B), the plasma BNP and Ang II levels in mice were augmented 2 weeks after TAC surgery. However, their plasma levels were effectively lowered by Andr treatment. In addition, the heart size and heart weight/body ratios were markedly enhanced in mice with TAC, which was mitigated after Andr administration (Figure 2(C,D)). Furthermore, Andr treatment in TAC mice led to a notable reduction in the cardiomyocyte cross-sectional size (Figure 2(D,E)).

### Andr attenuates cardiac fibrosis and reduces cardiac cell apoptosis in TAC mice

**Figure 3.**
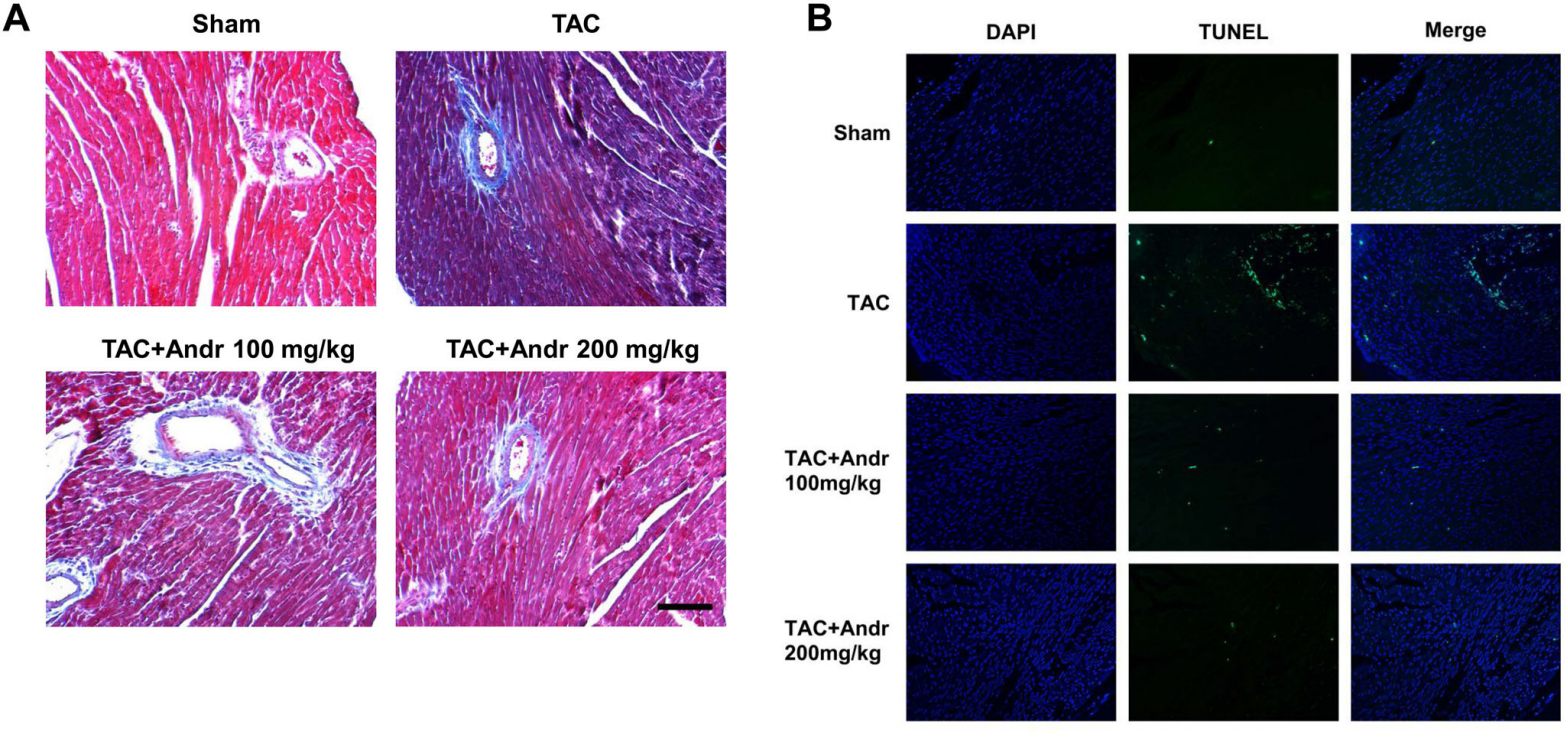
Andr attenuates cardiac fibrosis and reduces cardiac cell apoptosis in TAC mice. (A) Representative images of Masson’s trichrome staining of the hearts of mice in the sham, TAC, TAC + Andr 100 mg/kg and TAC Andr 200 mg/kg groups. Scale bar = 100 μm. Representative photomicrographs of TUNEL staining in the rat hearts. Scale bar = 20 μm.

Masson’s trichrome staining indicated that mice in the TAC group displayed a higher degree of cardiac interstitial and perivascular fibrosis than the sham group, which was weakened by Andr (Figure 3(A)). To determine the function of Andr on cardiac cell apoptosis in TAC mice, TUNEL staining of heart sections was performed. Results showed that TUNEL-positive cells in the TAC group was dramatically increased compared with the sham group, which was significantly decreased after Andr treatment (Figure 3(B)).

### Andr suppresses ang II-induced cardiac hypertrophy in vitro

**Figure 4.**
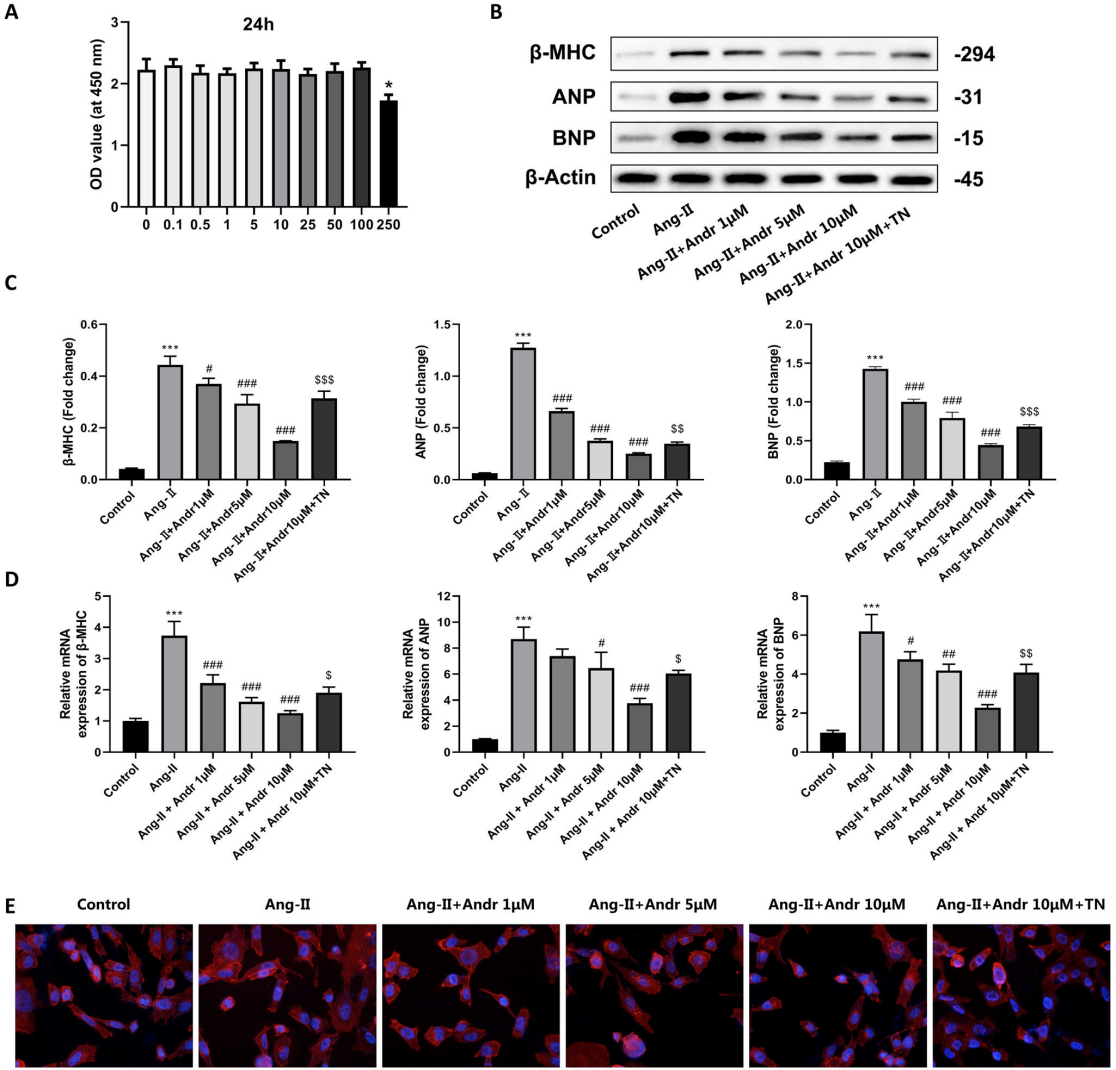
The effects of Andr on cardiac fibroblast *in vitro*. (A) Cell viability was accessed by the Cell Counting Kit-8 assay. (B–C) Western blot and (D) RT-PCR were performed to evaluate the protein and mRNA levels of β-myosin heavy chain (β-MHC), atrial natriuretic peptide (ANP) and brain natriuretic peptide (BNP) in cardiomyocytes in the indicated groups, respectively. (E) Immunofluorescence staining of F-actin and the cell surface area of cardiomyocytes in the indicated groups. The results are presented as a fold-change. ***p < 0.001 vs. the control group; ^#^*p* < 0.05, ^##^*p* < 0.01 and ^###^*p* < 0.001 vs. the Ang II group, ^$^*p* < 0.05, ^$$^*p* < 0.01 and ^$$$^*p* < 0.001 vs. the Ang II + Andr 10 μM group.

Next, the effect of Andr on cardiac hypertrophy in H9c2 cells was further investigated. H9c2 cells were stimulated with Ang II (1 mM) and treated with different concentrations of Andr (0, 0.1, 0.5, 1, 5, 10, 25, 50, 100 and 250 μM). CCK-8 results suggested that Andr administration did not affect H9c2 cell viability, except for 250 μM Andr (Figure 4(A)). PCR and Western blot results indicated that Ang II-treated H9c2 cells displayed upregulation of the protein and mRNA levels of hypertrophic markers ANP, BNP and β-MHC in a dose-dependent manner (Figure 4(B–D)), while Andr treatment significantly downregulated the expression of them both at protein and mRNA levels. As shown in Figure 4(E), Ang II stimulation for 48 h induced an increase in cardiomyocyte surface area linked to actin cytoskeleton reorganization as assessed by filamentous actin (F-actin) and was reversed in the presence of Andr. Besides, to illustrate that Andr can reduce myocardial hypertrophy by suppressing ER stress, we further treated cells with 10 μM of ER stress agonist TN + Ang II + 10 μM Andr and found that TN reverses the effect of Andr suppressed cardiac hypertrophy *in vitro* (Figure 4(B–D)).

### The ER stress inhibition of H9c2 cells by Andr

**Figure 5.**
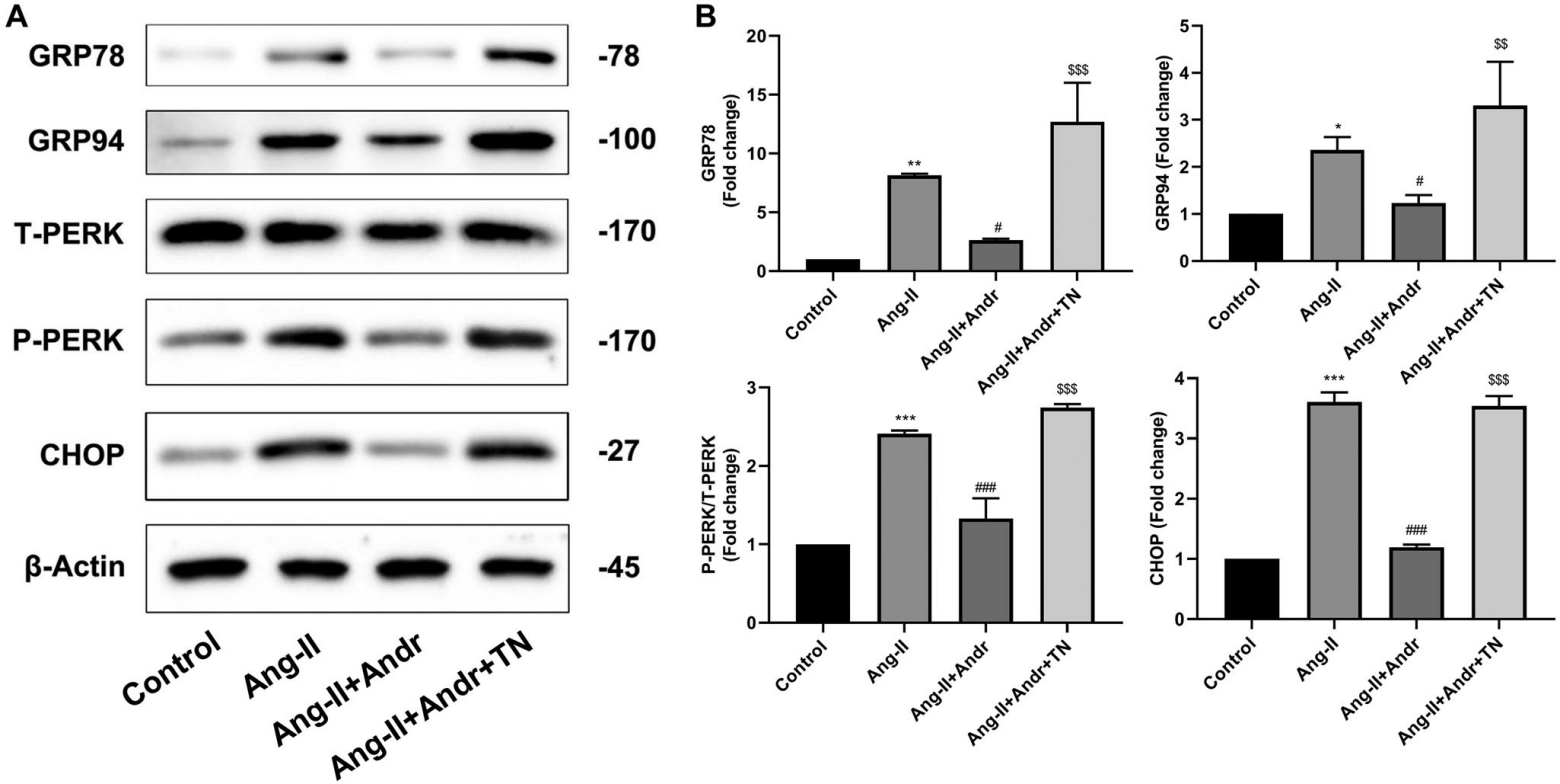
Andr inhibits ER stress in H9c2 cells. (A) Representative blots of GRP78, GRP94, total (T)-PERK, phosphorylated (p)-PERK and CHOP in control, Ang II, Ang II + Andr and Ang II + Andr + TN groups in H9c2 cardiomyocytes. (B) Qualifications of the Western blot assay. The results are presented as a fold change.**p < 0.01 and ***p < 0.001 vs. the control group; ^#^*p* < 0.05 and ^###^*p* < 0.001 vs. the Ang II group, ^$$^*p* < 0.01 and ^$$$^*p* < 0.001 vs. the Ang II + Andr group.

The critical role of ER stress in the development of cardiac hypertrophy is well known (Rani et al. 2017). Ang II stimulation significantly upregulated the protein expression of GRP78, GRP94, p-PERK and CHOP compared to the control group, while treatment with Andr resulted in a dose-dependent decrease in the protein expression of those sensors in H9c2 cells. But all these effects were reversed by ER stress agonist TN (Figure 5).

## Discussion

Although studies have shown that cardiac hypertrophy ameliorates myocardial pressure to some extent, it can lead to irreversible functional deterioration and eventually heart failure if it persists (Zhang et al. 2019). Establishing an animal model similar to human disease is a crucial step in the treatment of cardiac hypertrophy. Currently, there are many methods for modelling cardiac hypertrophy in mice clinically, and chemical drug induction and physical operation modelling like the pressure overload heart failure model, the volume overload heart failure model and the post-myocardial infarction heart failure model are commonly used (Chen et al. 2019; Bosch et al. 2021). However, chemical induction is less successful and more time consuming and physical operations can trigger low survival. To understand the mechanisms of pressure overload-induced myocardial atrophy, the transverse aortic constriction (TAC) model was developed (Rockman et al. 1991). Constriction of the aortic arch in animals will increase the resistance of the left heart to expel blood, resulting in increased cardiac work and oxygen consumption, which will lead to myocardial hypertrophy to heart failure, making heart failure from compensation to decompensation due to excessive pressure loading, hence a good correlation to clinical cardiovascular disease (Xiao-Mei et al. 2008). Additionally, this model does not cause a sudden increase in left ventricular afterload and is a short, minimally invasive procedure with a high postoperative survival rate, which can better simulate the evolution of left ventricular hypertrophy due to pressure overload (Faerber et al. 2011). In this study, the mice model of TAC model was successfully established to detect the impact of Andr on cardiac hypertrophy and we found that Andr improved left ventricular systolic dysfunction, attenuated cardiac hypertrophy and fibrosis and reduced cardiac cell apoptosis in TAC mice. Also, we observed that Andr ameliorated Ang II-induced hypertrophic response and enhanced cardiomyocyte size in H9c2 cardiomyocytes by suppressing the activation of ER stress-related markers (GRP78, GRP94, p-PERK and CHOP).

Andr has been reported to possess beneficial properties, including immune regulation, anti-inflammation, anti-hyperlipidaemia and anti-oxidation (Wang et al. 2014; Lim et al. 2016; Yuan et al. 2016). In recent years, the protective effect of Andr on cardiac function has attracted the attention of researchers worldwide (Wu et al. 2017). However, its efficacy on pathological hypertrophy and fibrosis has not been determined *in vivo*. In the present study, M-mode echocardiography and left ventricular mass index revealed that mice in the TAC group exhibited significant deterioration in left ventricular systolic function two weeks after TAC surgery, indicating that TAC induced cardiac dysfunction in mice. While Andr dose-dependently decreased LVESD and LVEDD and enhanced left ventricular EF and FS, suggesting that it ameliorated left ventricular contractile function. Pathological cardiac hypertrophy is an augmentation in cardiac mass as myocardial cells increase (Dolinsky et al. 2013). Compared with the sham group, the heart size, heart weight/body ratio and cardiomyocyte cross-sectional size in TAC mice were remarkably elevated, while Andr restored cardiac wall thickness almost to nearly the sham level. Meanwhile, the ELISA assay indicated that the levels of hypertrophy markers (BNP and Ang II) in TAC mice were higher than that in normotensive mice, whereas the presence of Andr rescued these changes. Additionally, our *in vitro* study illuminated that Andr administration lowered the upregulation of the protein and mRNA expression of ANP, BNP and β-MHC induced by Ang II administration. Therefore, Andr represented a potential agent for the attenuation of cardiac hypertrophy.

Cardiac fibrosis, a typical feature of hypertrophic cardiomyopathy, occurs as a compensatory response to overcome enhanced cardiac wall stress induced by various stimuli, and the resulting pathological changes can culminate in ventricular dysfunction, cardiomyocyte hypertrophy and apoptosis (Baudino et al. 2006). Recent research has elucidated that the proliferation of mesenchymal stem cell-like cells in cardiac perivascular regions after the cardiac injury can generate myofibroblasts that contribute to cardiac fibrosis (Travers et al. 2016). Consistent with the characteristics of cardiac fibrosis described above, Masson’s trichrome staining showed that cardiac fibrosis in TAC mice was characterized by obvious interstitial and perivascular fibrosis, which might be associated with enhanced cardiomyocyte apoptosis. And this was then alleviated after Andr treatment. Furthermore, our *in vitro* results noted that Ang II stimulation led to F-actin reorganization, and the early induction of such cytoskeletal response has been demonstrated in mice cardiomyocytes exposed to a variety of hypertrophic stimuli. In comparison to the model group, Andr dose-dependently blunted the pro-hypertrophic effect of Ang II, as evidenced by a reduction in cell surface area.

When UPR is unable to compete with severe and prolonged external stimuli, ER stress sensors are triggered to induce cell apoptosis (Lei et al. 2017). A previous study demonstrated that ER stress is linked to the pathogenesis of cardiac hypertrophy, which increases the risk of heart failure (Rani et al. 2017). Since the underlying mechanism of Andr’s cardio-protective function remains unclear, we speculated that Andr regulates apoptosis of cardiomyocytes by modulating ER stress. To verify the idea, Western blot was performed to detect the expression of PERK, GRP78, GRP94 and CHOP. Earlier research suggested that ER-located chaperones GRP78 and GRP94 can bind to transmembrane proteins such as PERK and remain inactive under normal physiological conditions (Wu et al. 2020); however, GRP78 can maintain the internal environment of the tumour under ER stress conditions, which improves cancer cell survival (Li et al. 2015). And CHOP, a basic leucine zipper-containing transcription factor regulated by ATF6 and PERK pathways, induces cell apoptosis by indirectly activating Bim mRNA expression during ER stress (Puthalakath et al. 2007). Our *in vitro* results revealed that Ang II stimulation significantly upregulated the protein expression of GRP78, GRP94, p-PERK and CHOP, while 10 μM Andr treatment led to a dose-dependent reduction in the protein expression of these ER-related markers in H9c2 cells. Strikingly, all these effects were reversed by 10 μM ER stress agonist TN. Taken together, these findings suggested that Andr abated cardiac hypertrophy by suppressing ER stress.

## Conclusions

The present study indicates that Andr can alleviate cardiac dysfunction and cell apoptosis and abate cardiac hypertrophy and fibrosis in a dose-dependent manner. Furthermore, we document the effective suppression of ER stress by Andr treatment in cardiomyocytes and reveal that ER stress-related markers mediate the anti-hypertrophic effect of Andr, suggesting that Andr has the potential of to be a novel agent for the treatment of cardiac hypertrophy.

## Data Availability

The data used to support the findings of this study are included in the article.
